# Nursing Students' and Preceptors' Experiences with Using an Assessment Tool for Feedback and Reflection in Supervision of Clinical Skills: A Qualitative Pilot Study

**DOI:** 10.1155/2021/5551662

**Published:** 2021-05-18

**Authors:** Hilde Plathe, Elisabeth Solheim, Hilde Eide

**Affiliations:** Faculty of Health and Social Sciences, Department of Nursing and Health Sciences, University of South-East Norway, PoBox 7053, N-3007 Drammen, Norway

## Abstract

**Background:**

There is a need to improve students' learning in clinical practice. Undergraduate students need guidance when it comes to transferring knowledge from the classroom to clinical practice in community health services. Competence Development of Practical Procedures (COPPs), a simulation assessment tool, was used to explore students' and preceptors' experiences with feedback and reflection during the supervision of clinical skills in real practice.

**Method:**

This was a pilot study with a qualitative exploratory and descriptive research design. Four students in their first year of a bachelor's programme in nursing and four preceptors participated. Data were collected from eight clinical skills performance assessments, audio recordings of supervision, and open-ended questionnaires. Data were systematized, categorized, and analysed using qualitative content analysis. *Findings*. Participants' experiences were divided into five categories: “learning environment, an atmosphere of respect, acceptance, and encouragement,” “students' reflections on their own personal learning,” “students' reflections on various care situations,” and “students' and preceptors' assessment and feedback.” Participants found COPPs easy to use and providing structure for assessment, feedback, and reflection during supervision. Concepts related to learning clinical skills became visible for both students and preceptors and helped students assess their performance of clinical skills. Through verbalization and reflection in supervision, participants established a consensus around what students knew and what they needed to learn.

**Conclusions:**

The students and preceptors experienced the tool as a supportive structure to enhance feedback and reflection for the learning of clinical skills in municipal healthcare services. COPPs filled a gap in practice by providing a language for students and preceptors to articulate their knowledge and increasing students' awareness of what constitutes a good performance. The tool supported the coherence of concepts, enhanced clinical reasoning, and promoted deeper thinking and reflection, and the students gained insight into their own needs related to learning clinical skills.

## 1. Introduction

Nursing is a practice-based discipline, and clinical placement is a vital part of nursing education in bachelor's programmes. In Norway, the bachelor's degree programme in nursing runs over three years. In accordance with the EU's requirements, 50 per cent of the study time is reserved for clinical practice, either in hospitals or in the municipal health service [1].

However, newly graduated nurses demonstrate a lack of expertise in clinical skills [2]. Patients often have complex disorders and challenges, which can make it difficult for novices to learn to think like a nurse [3, 4]. Clinical skills are complex, requiring technical expertise, theoretical and practical knowledge, caring intention attuned to both the patient and environment, and ethical consideration [5, 6]. Before, during, and after clinical skill training, students and nurses must conduct a number of clinical skill assessments based on a process of clinical reasoning. One study showed that nurses engaged in up to 50 significant instances of clinical reasoning in one eight-hour shift in a medical admissions unit [7]. Clinical reasoning is defined as the processes by which nurses and other clinicians make judgments, including the process of generating alternatives and choosing the most appropriate one(s) [4].

Novice and expert nurses often have different cognitive thinking strategies [8]. Novices require more time and training to reach a higher level of clinical reasoning and judgment. The primary reasons for adverse patient outcomes are failure to properly diagnose, failure to implement appropriate treatment, and inappropriate management of complications [8]. Learning to think like a nurse is an important component of clinical practice [4]. Students need support as well as practical training to become “fit for practice” and transfer learning from the classroom to their practice as nurses [9, 10]. Clinical supervision aims to assist students in applying the theory of nursing in real-life situations and in integrating theoretical knowledge and clinical skills, and it is essential to ensuring that nursing students can provide safe and competent care before they graduate [11].

In clinical practice in homecare and in nursing homes in Norway, students are traditionally supervised by a preceptor. The preceptors in this study were nursing staff, including both registered nurses (RNs) with a bachelor's degree and practical nurses (PNs) with a vocational degree. The concepts of preceptor, clinical supervisor, and mentor are defined differently in the literature [12, 13]. A preceptor has more experience and can help less-experienced students reach their learning potential [13] and achieve learning outcomes in clinical practice. This ability to achieve desired outcomes with assistance from more experienced individuals has been termed the “zone of proximal development” [14].

Educational practices must help students engage with patients to identify areas that need improvement, ideally in a debriefing with preceptors who can provide feedback and help students develop insight into their own clinical thinking [4]. Indeed, it has been argued that feedback is the most effective strategy for making learning visible to students [15]. Effective learning in clinical practice also requires that students have a broad experience base and an opportunity to reflect on and analyse the situations in which they are involved [11]. Reflection is often understood as looking back or looking at, as in “reflection on action” and “reflection in action” [4, 16], but it can also be looking forward (i.e., “feed forward”) or “reflection beyond action” [17, 18]. As action and reflection are closely linked elements [4], reflecting on action means thinking about what one is doing while one is doing it. Systematic reflection on action will increase learning and further develop competence [19]. Without systematic reflection, learning will occur, but it will be random and may be deficient [20].

Nurses are required to supervise students as part of the nursing job. The challenge is that many nurse preceptors have a lot of tacit knowledge, which is implicit knowledge that is based on lived experience and cannot be codified [21]. To address this challenge, the authors of this article wanted to test a feedback and reflection tool designed to support supervision of students' learning of clinical skills in a simulation centre at a Norwegian university. The tool, Competence Development of Practical Procedures (COPPs), can be used to assess the performance of many clinical skills [22]. COPPs was developed from and inspired by the Model of Practical Skill Performance [5], person-centred practice in nursing [23], updated online guidelines in healthcare and clinic-based knowledge [24, 25], and nursing student syllabi and the power of feedback [26]. COPPs (Appendix 1) provides a structure for reflection and feedback and makes visible the complexity of learning clinical skills involving technical and theoretical aspects and relationships with patients. The tool is divided into three areas: (1) preparation, planning, performance, and supplementary work, (2) overall assessment, and (3) knowledge of clinical skills. It also includes the performance of clinical skills to be assessed as “excellent,” “partially completed,” or “missing” and a column for writing additional comments. This is a formative assessment that provides a structure for feedback and reflection on learning clinical skills in high-fidelity simulations [22]. COPPs is designed to provide structure for student learning related to clinical skills, for peer assessment, and for in-depth feedback on the learning process from teachers. However, the tool may also have the potential to support feedback and reflection in supervision of students by preceptors in real practice.

### 1.1. Aim

The aim of this pilot study was to explore students' and preceptors' experiences of using COPPs as a tool for supporting feedback and reflection during supervision of clinical skills in real practice.

## 2. Methods

This pilot study had a qualitative approach with an exploratory and descriptive design [27]. In this context, a qualitative approach enabled a focus on specific aspects of meaning and the experiences of selected participants. An exploratory design is appropriate when there is little known about the phenomenon under study, as is the case in the present study. Finally, a descriptive design was used to describe the characteristics of students' and preceptors' experiences using the COPPs assessment tool in supervision and to provide the reader with a clear, accurate picture of the situation.

### 2.1. Settings and Participants

This pilot study was carried out during spring 2017, at the end of the students' first year in the bachelor's programme, and during their first clinical placement. They spent eight weeks either in a nursing home or in homecare. Patients in these settings are characterized by multimorbidity, polypharmacy, and/or cognitive impairment.

Four nursing students and four preceptors volunteered to participate in spring 2017 (Table 1). All participants were women. The two homecare nurses nursing were recent RN graduates. They had completed a five-hour educational course in supervision at the university, and one of them had recently begun further training in supervision (30 ECT). The two preceptors in the nursing homes were PNs; both had extensive professional experience, but one of them had never supervised students before.

### 2.2. Procedures and Data Collection

The authors provided oral information to the department administrators before the students' clinical practice. Two randomly selected municipal health services that had supervisory responsibility for students in the bachelor's programme in nursing at the university were invited to participate in this pilot study. Out of fifteen students and their preceptors who were asked to participate, four students and their preceptors volunteered to participate. The students were familiar with COPPs from their simulated clinical skill training at the university. The preceptors received written information about the study plan, the students' learning outcomes, and COPPs before the clinical period. The academic staff from the university provided preceptors with information and presented the study at the first meeting with the students and the preceptors. The researchers were not in contact with participants during the study.

**Figure 1:**
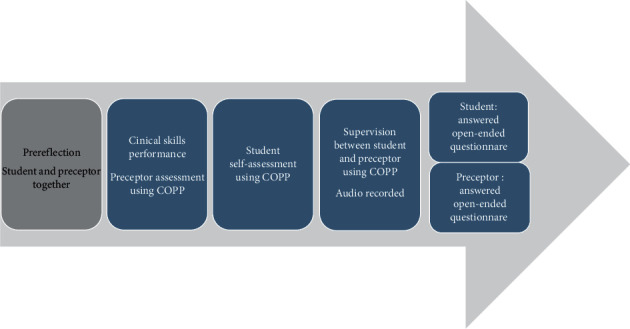
Data collection and supervision of one student's clinical skill.

Each student performed two clinical skills in this study. The first clinical skill, selected by the researchers, involved students caring for a patient that needed “personal hygiene” assistance. The second, selected by the student, consisted of either measuring blood sugar or performing a subcutaneous injection.  Figure 1 provides an overview of the data collection and supervision process related to one clinical skill.

COPPs gives a structure for performance of clinical skills and was used for reflection before action and to help the students and preceptors make a plan and discuss the concepts in the tool. As the student performed the clinical skill, the preceptor used the tool for observation and assessment and evaluated the student by ticking the appropriate box and adding comments where applicable. Shortly after performing the skill, the students assessed themselves using COPPs. Data collected for one clinical skill at this stage consisted of two completed COPPs, one from the student and one from the preceptor.

Each student and preceptor then used the completed tool in supervision to reflect together. This took place in a suitable room. The student was responsible for audio recording the dialogue to enable access to nonverbal and verbal elements along with communication cues [27]. After supervision, each student and preceptor completed a questionnaire with eight short, open-ended questions (Appendix 2). Open-ended questions allowed the participants to answer freely and spontaneously [27]. These were used to gain a deeper understanding of each student's and preceptor's experiences using COPPs. The data collected for all participants consisted of 16 completed tools (8 from the students and 8 from the preceptors), 8 audio-recorded debriefings, and 16 answered questions (8 from the students and 8 from the preceptors). The data were collected by the researchers shortly after students' clinical practice, and there was no relationship between researchers and participants during data collection.

### 2.3. Analysis

Data were systematized, categorized, and analysed using qualitative content analysis [28]. Qualitative content analysis emphasizes the linguistic, inductive, or text-driven search for patterns; in this study, the analysis was carried out in four steps.

Step 1: the authors listened to the audio recordings several times and transcribed them. Next, they read the transcribed text and completed COPPs systematically. Having gained a comprehensive impression of the data, the researchers then discussed the material and identified ‘meaning units.' This step was inductive, with a low degree of interpretation at the textual level. Step 2: meaning units were further condensed and coded to organize the material; these units were derived through an inductive process and understood in relation to context. Codes emerged as data that seemed to cluster as a result of the condensing in the first step. Different codes were compared to the transcribed text and were interpreted in light of the study's aim. This interpretation consisted of moving between the whole and the part in what is described as a hermeneutic circle [29]. Step 3: codes were abstracted into broader categories. A category is an abstraction of condensed text that is interpreted in light of the researchers` own learning, one's own experience, and the researcher's comprehension, shaping the overall understanding and interpretation of the material. A comparison of the codes identified similarities and differences that were consolidated into categories and subcategories. Step 4**:** the data were further analysed, and categories and subcategories from all participants were compared. New dimensions emerged, and new subcategories were created. Through this extensive analytical work involving reflection on the meaning of participants' stories, new subcategories were created and four main categories were identified.  Table 2 shows an example of this process. To strengthen trustworthiness and reach consensus, the authors discussed and reflected on the data at all steps of the analysis.

#### 2.3.1. Open-Ended Questions

The responses from each of the students and each of the preceptors to the open-ended questions in the questionnaire were transcribed by the authors. They were then systematized for all students and all preceptors in an attempt to identify similarities and variations.

### 2.4. Research Ethics

The head of the Faculty of Health and Social Sciences at the University of South-Eastern Norway gave permission for the study. Participation in the study was voluntary; the participants were informed of the study design, provided written consent, and were free to withdraw at any time. The study was classified as an educational evaluation: no patients were involved and, therefore, no ethics committee approval was required. The Norwegian Social Science Data Service approved this study in February 2017 (53190). The notes and audio files were scanned immediately and stored securely, and the data were anonymized.

## 3. Findings

Five main categories emerged based from the qualitative data analyses: “learning environment, an atmosphere of respect, acceptance, and encouragement,” “students' reflection on their own personal learning,” “students' reflection on various patient-care situations,” “students' and preceptors' assessment and feedback,” and “students' and preceptors' experiences of using COPPs in clinical practice.” The participants' own expressions are highlighted below, with reference to the numbers and letters from Table 1. A summary of the open-ended questions follows the presentation of the findings.

### 3.1. “Learning Environment, an Atmosphere of Respect, Acceptance, and Encouragement”

Students and preceptors met for supervision and used COOP to discuss and assess discussion the students' performance. The results revealed that all the students were open to sharing their experience about practicing skills in various patient-care situations using COPPs. They were sometimes concerned about the quality of their care in personal hygiene and believed that patients may have noticed their lack of confidence. Students talked openly about what they had missed. One student recounted, “I saw the toe, but I do not know anything about nail care. So I chose not to do anything.” In supervision, this student openly shared her incompetence in principles of hygiene. The preceptor accepted this openness and acknowledged the student by saying “I understand” or making small utterances like “Yes” or “Mm” to affirm statements the student made.

The overall tone in supervision was friendly, calm, and pleasant: students and preceptors showed each other respect through their honest communication using COPPs. The participants used the tool systematically as a guide to structure their conversations about clinical skills. Specific goals and learning outcomes in COPPs were consistently included in their communication. For example, regarding “overall assessment,” one student said, “It was done with a mix of fluency, without hesitation and unnecessary breaks. I think it was excellent and without hesitation and with ease” (1). The preceptor (a) responded straightforwardly: “You were empathic and used nonverbal communication when the patient was unsure. I think it was excellent.” Honest communication helped this student and preceptor to clearly visualize concepts using the assessment tool to find shared meaning in a safe learning environment during the supervision.

There were some complex and challenging care situations involving patients. The students expressed their experiences of lacking “Knowledge of clinical skills” related to indications, complications, observation, documentation, and ethical challenges. In supervision, the preceptors were able to deepen students' knowledge related to the patient's situation and the context. The students were honest about their weaknesses, specifically about being unsure how to behave and communicate with patients, and expressed surprise about unexpected patient-care situations. Nevertheless, two students (1 and 4) could not identify any ethical challenges. Later, in supervision with the preceptor, they decided to learn more about ethics in relation to clinical patient-care skills.

The preceptors were sometimes concerned about the patients' security during students' performance of clinical skills; in these cases, they ticked “missing” under “performing procedure according to updated guidelines” in COPPs. However, when telling students that improvement was needed, their tone of voice remained calm. The students responded to the preceptors' instruction with words and silence, sometimes followed by a shared laugh, which seemed positive for both.

One of the preceptors (d) used concepts in COPPs to ask questions that invited the student to elaborate, summarize, conclude, or move on, including “What do you think you could do differently, then?” (missed hygiene); “Do you have anything else to add?” (lack of knowledge about complications, observations, and ethical challenges); “Tell me more about this” (missed introduction); or “Can you sum up?” (about the topic). It should be noted that this preceptor had the most formal pedagogical training in supervision.

COPPs as a structure for the supervision team seems to be a safe and predictable framework for the students and preceptors, which provided a good atmosphere characterized by respect, acceptance, and encouragement.

### 3.2. “Students' Reflection on Their Own Personal Learning”

The students reflected on their own personal learning when responding to concepts in COPPs. They highlighted “Knowledge of clinical skills” in particular during supervision. One of the students stressed the importance of observations: “I feel I have become better at doing observations and not just doing the procedures” (1). This student learned about assessing observations while caring for a real patient. Another student, who helped a patient who needed a subcutaneous injection, reflected “It is not quite the same on humans as it is on dolls” (3). This student discovered a gap between learning through simulation in a lab setting using dolls and learning in clinical practice.

The students reflected on the emotions involved in learning. Many felt uncertain, somewhat anxious and hesitant, and inexperienced enough not to feel completely confident. However, this varied from student to student depending on their earlier experiences with patients, clinical skills, and healthcare contexts. One student in homecare nursing said, “I have not helped so many people with hygiene during the evening shift here. Therefore, I am a bit unsure how to do it” (2). Another student managed her situation with ease because she had experience with the patient and felt confident with this clinical skill: “I think I am safe in the situation. I know how to perform the clinical skill, and I know why. I feel I can tell the patient what I know” (4). This student's experience helped her provide safe care to the patient during the subcutaneous injection and deepened her knowledge of clinical skill in supervision.

### 3.3. “Students' Reflection on Various Patient-Care Situations”

The students cared for patients in homecare and nursing home settings. Patients with multimorbidity had cognitive impairment and the students were not fully prepared to handle such patient situations. During clinical skills supervision, one student reflected, “It is difficult to prepare for introducing yourself and checking ID” (1). Another student noted, “Communication with this patient is difficult for me” (2). Using COPPs to reflect on ‘overall assessment' situations with elderly patients, particularly those suffering from dementia, enabled novices to use appropriate communication during clinical skills. This reflection became visible for both the student and preceptor during supervision.

Personal hygiene was challenging for all students at this level of first clinical practice. One student reflected, “I've never cared for this patient before. After all, it is a challenge to get to know the patient. I asked the patient, but I think it went very well. To get to know her better, I asked her a lot about what she wanted to do herself and if she had any routines” (3). Supervision involves helping students prepare for and perform clinical skills through dialogue and reflection. This student asked the patient many questions, but in the supervision, this student realized there was a need for better preparation and planning to perform a clinical skill.

Two students chose “subcutaneous injection” for their clinical skill supervision. One student reflected, “I chose to put the syringe at 45° instead of 90°. She is of normal weight so I could have set it at 90°, but her skin was so thin” (4). This student had mastered the technical aspect of a clinical skill and deepened their individual knowledge, in addition to clarifying individual goals and assessing the quality of their performance in supervision using COPPs. Supervision is about clarifying each student's individual goal and the support they need to take responsibility for nursing interventions or clinical skills.

### 3.4. “Students' and Preceptors' Assessment and Feedback”

Students used COPPs to assess themselves shortly after performing a clinical skill and before supervision. They rated themselves “excellent” or “partially completed” in “preparation and planning” and “overall assessment.” The students' self-assessment with regard to “knowledge of clinical skills” ranged from “excellent” to “missing” for two students. On the other hand, two of the students did not use this part of COPPs at all, but, instead, indicated that they would wait for supervision with their preceptor. One novice student was not certain about ‘knowledge of clinical skills' and wrote, “I don't know what to say…. This is more of something we are supposed to do together..., indications or purpose of the procedure” (2). In other words, the student's knowledge of clinical skills was limited and needed to be developed in the supervision.

The preceptors' assessments of students' performance of clinical skills ranged from “excellent” to “missing.” All the preceptors actively used the additional comments column. They wrote things like “no plastic aprons,” “student asks the patient too much,” “somewhat uncertain due to the situation,” and “helped student because she had not performed this procedure.” They then used these notes to structure their input in supervision.

One of the preceptors noted shortcomings: “I have written missing. You did not introduce yourself. You did not ask if this is the right patient in front of you” (b). This student neglected to ensure patient safety before the subcutaneous injection. In the supervision, the preceptor pointed out the student's lack of knowledge and responsibility. Another preceptor also provided clear feed-forward messages during supervision: “Continue to work on this and manage more injections. It is something you need to do a bit more” (c). Effective assessment and feedback using the tool made learning potential visible.

In addition to having the opportunity to point out what was deficient and needed improvement, the preceptors were also able to use the tool to highlight what they found excellent. This constructive dialogue during supervision, therefore, bolstered a positive experience of a shared sense of direction.

### 3.5. “Students' and Preceptors' Experiences of Using COPPs in Clinical Practice”

In the following, data from the open-ended questions (Appendix 2) answered by students are summarized first, followed by the preceptors' responses.

Students reported being familiar with the tool from simulations at the university's lab where they had used it to self-assess clinical skills and conduct peer assessment. In the present study, students experienced that the tool was appropriate to use for self-assessment in real practice as it helped them be aware of their own actions, proposed concepts to systematize their performance, and made it easier to articulate what they still needed to learn. In the students' conversations with their preceptors in supervision, all three columns (“excellent,” “partially completed,” and “missing”) were helpful in providing both feedback and feed forward. Students reported that the preceptors allowed them to be active in assessing their own performance. Together, students and preceptors systematically compared the completed COPPs as a way of structuring conversation about the students' strengths and weaknesses in performance, reflecting on and deepening their knowledge of relevant aspects such as hygiene, overall assessment, and knowledge of clinical skills.

Preceptors reported that although COPPs was new for them, it was easy to use, and they used all of the categories and subcategories, elaborating on them if needed during the supervision. COPPs enabled them to give precise feedback and feed forward, and it highlighted many aspects of the students' performance. A nurse wrote, “The tool made me more aware of everyday procedures such as personal hygiene” (d). Preceptors, who have more experience than students, may need to articulate tacit knowledge related to basic procedures and COPPs can make concepts visible and help them articulate this.

In assessing the students, the preceptors emphasized criteria related to the students' “excellent” performance regarding care of the patient, caring comportment, and proper planning to ensure patient security. Preceptors found it useful to write comments and tick the appropriate boxes next to the concepts, and they used these notes when providing feedback during supervision. Preceptors noted that they first asked students about their self-assessments in COPPs before discussing further and deepening knowledge together. One nurse (c) noted the importance of highlighting what was missing in the student's performance with regard to taking care of the patient, thus helping the student understand how to improve.

## 4. Discussion

The aim of this pilot study was to explore students' and preceptors' experiences using COPPs to structure feedback and reflection in clinical skill supervision in real practice. The major findings reveal that students and preceptors found the tool useful for structuring supervision and learning of clinical skills in their first practice in community health services.

### 4.1. Visible Learning

A robust conceptual framework and good feedback practices are important for successful formative assessment [30]. Educational assessment that places the student at the centre of the assessment process helps make learning visible [18, 26]. Clinical skills are complex and based on both theoretical and practical knowledge; they also require communication skills and ethical and moral considerations, all of which must be tailored to the individual patient's needs [5, 31]. COPPs identifies and systematizes the theoretical and practical concepts that are applicable to feedback and reflection on different clinical skills [22]. There are other similar tools or frameworks used to structure reflection and supervision. The Model of Practical Skill Performance [5, 32] has been used at some nursing schools in Norway. While we think this is a good model, we found that students needed a tool that was more concrete and specifically tailored to learning practical skills and associated knowledge.

An assessment tool like COPPs can provide a shared language for articulating and even transforming knowledge and increasing competence [33]. Students in this study used COPPs to assess themselves before supervision with preceptors, which allowed them to gain insight into their own learning needs and to visualize concepts [26]. Their self-scores and comments provided information about students' strengths and weaknesses, highlighting the gap between what they were supposed to know and what they actually knew.

### 4.2. Tool for Enhanced Reflection and Feedback in Clinical Practice

Students often feel vulnerable when having their novice knowledge and practice assessed. COPPs can provide a safe and familiar framework for assessing learning goals. This study showed the atmosphere between the participants was characterized by respect, acceptance, and encouragement.

There is a lack of critical reflection among students [34]. Because of embodied knowledge, nurses and students find it difficult to verbalize thoughts and explain their cognitive processes [35, 36]. Results from our study show that COPPs may be helpful as a reflection and assessment tool in supervision of clinical skills. Concrete and constructive feedback from an experienced preceptor is important in formative assessment. This is in line with the idea of the “proximal zone” [14], in which students must be supervised by someone more competent. Verbalizing and reflecting cognitively on the actions they perform helps students improve clinical reasoning skills [8].

Strengthening cognitive skills, such as discussion and reflection, strategies, planning, analysis, and self-assessment, seems to be an effective and robust approach to learning clinical skills [30, 35]. An important goal of most clinical skill assessment or supervision tools is to make students aware of what constitutes a good performance [32]. However, students who focus only on the steps being performed demonstrate a lower level of competence when performing skills than those who are involved in discussion and systematic thinking in parallel with their training [37]. This may be because an advanced level of understanding is necessary to recognize the complexity of clinical skills [38].

In this study, students and preceptors used COPPs as a starting point and framework to underpin discussion and reflection that elicited deeper thinking. It has been argued that “knowing that” (theory) is essential to describing and providing reasons for “knowing how” (practice) in developing nursing skills and knowledge [39]. A novice is a newcomer who has little or no experience in handling clinical skills in real-life situations. Through reflection and assessment using the common concepts in COPPs, students engage in a process that, over time, enables them to acquire a higher level of the analytic skills needed for clinical reasoning.

Previous studies highlight a lack of pedagogical competence among preceptors [11, 40]. Students, therefore, often experience a lack of supervision and professional dialogue with preceptors who could help them link theory and practice [41]. To help students achieve a professional standard in clinical skills, it has been recommended that preceptors be nurses with pedagogical education in supervision [35]. Our study shows that preceptors' competences vary, and this seems to influence the quality of the supervision. Despite these differences, COPPs appeared to support student-preceptor interactions by establishing shared concepts and meanings and by structuring the preceptors' guidance [22, 42]. Formalized strategies or educational models such as this one have been shown to be necessary to enhance students' learning experiences [43].

### 4.3. Coherence of Concepts: Bridging the Gap?

To bridge the gap between theory and practice, there is a need for coherence between the theoretical approaches used in the classroom and the approaches used in clinical practice so that students ‘speak the same language' at university and in clinical settings [44]. The primary goal of professional education is to bridge this gap [35]. COPPs may help with this as it provides students and preceptors with concepts that are common in both university and clinical settings.

The concept of coherence is closely related to meaning [45]. COPPs helps students and preceptors create “a common meaning between minds” and provides common concepts and a framework for communication in supervision [21, 33, 39]. Knowledge might transfer more easily between university and clinical practice if students and teachers maintain close connections and working links with practitioners [46]. COPPs provides a structure for supervision, and the concepts are flexible enough that they can be adapted to different contexts in clinical practice. Knowledge translation is a process that takes time, and it is enhanced by appropriate support and formative assessment from preceptors [47]. COPPs supports this process by providing structure and shared concepts for both students and preceptors.

### 4.4. Methodological Considerations

To strengthen the credibility of this pilot study, participants were randomly selected from two different municipal health services. Four students and their preceptors participated, all women. A different recruiting process for students might have included more participants with varied demographic. Transferability is difficult to achieve in qualitative studies because the focus is on acquiring deeper knowledge and samples are small [48]. To compensate for this, we used different methods of data collection: completed COPPs, audio recordings of supervision, and questionnaires with open-ended questions to improve readability and flow. This resulted in rich, expansive, and varied data that provided insight related to the aim of the study.

Both authors conducted and transcribed the data. To enhance the quality of the analyses, all the investigators discussed the results and reached a consensus. Credibility was established by selecting the most appropriate meaning units, categories, and themes to cover the data. Dependability was strengthened by using a coding list to prevent changes in meaning between the coding and decoding process. Validity was strengthened by the researchers' self-reflection on their role as teachers with a professional nursing background and expertise in the field.

A weakness of this study is that only the students were familiar with COPPs as a tool for feedback and reflection. If the preceptors had been accustomed to using COPPs, they would likely have more actively used it for preparation and planning during the students' clinical practice. A second limitation is that the findings of this pilot study are limited to one university in Norway and are, therefore, specific to both location and context.

## 5. Conclusions

The results indicate that the participants found that COPPs provided support and structure for feedback and reflection in clinical reasoning and clinical skills development in municipal healthcare services.

An important goal of COPPs as a tool for assessing clinical skills is to make students aware of what constitutes a good performance. In a practice that is characterized by clinical skills and tacit knowledge, COPPs seems to provide a language for students and preceptors to articulate their knowledge and competence. Through structured reflection and assessments, the students revealed their own strengths or weakness and got insight into their own learning needs. COPPs seemed to support the transfer of knowledge and helped bridge the gap between university and clinical practice. The tool supported the coherence of concepts, enhanced clinical reasoning, and promoted deeper thinking and reflection when learning clinical skills. This was a pilot study, and further studies are needed to evaluate this tool with a broader sample and/or in other contexts of clinical practice in nursing education.

## Figures and Tables

**Table 1:** Students (1–4) and preceptors (a–d) together in different contexts.

Student				Preceptor			
	Age	Context		Age	Education	Nursing experience	Number of earlier student supervisions
1	21–25	Nursing home	a	>55	Practical nurse	34 years	0
2	20	Nursing home	b	>55	Practical nurse	30 years	6–10
3	21–25	Homecare nursing	c	20–25	Registered nurse Course in supervision, commenced education in supervision	4 years	6–10
4	20	Homecare nursing	d	20–25	Registered nurse Course in supervision	3 years	6–10

**Table 2:** Example of the analytical process.

Meaning unit	Condensed meaning unit	Code	Subcategory	Category
I do not think I introduced myself. I Knew it was him... Oh, I forgot to introduce myself…	Oh, I forgot to introduce myself…	Student's self-assessment	Clarify goal	Students' and preceptors' assessment and feedback
I have written missing. You could have been a bit clearer to the patient about who you are and what you were going to do. I have written missing.	You could have been a bit clearer. I have written missing	Preceptor's assessment	Feed forward	
I think it was excellent that you observed so much about everything from feet to skin and how the patient felt. It was excellent to observe.	Observation was excellent from feet to skin patient's feelings	Preceptor's assessment	Feedback	

## Data Availability

The data from notes, audio-recorded interviews, and questionnaires used to support the findings of this study are available from the corresponding author upon request.

## Supplementary Materials

Supplementary Materials. Appendix 1: the assessment tool Competence Development of Practical Procedures (COPPs). Appendix 2: questionnaire to preceptors and students.
