# Supplementary material for: Nursing Students' and Preceptors' Experiences with Using an Assessment Tool for Feedback and Reflection in Supervision of Clinical Skills: A Qualitative Pilot Study
Source: Nurs Res Pract. 2021 May 18;2021:5551662. doi: 10.1155/2021/5551662 (PMC8154278; doi:10.1155/2021/5551662)
Supplement: Supplementary Materials. — Appendix 1: the assessment tool Competence Development of Practical Procedures (COPPs). Appendix 2: questionnaire to preceptors and students. [file 5551662.f1.zip › 5551662.f1/Appendix 1, Competence development of Practical Procedures (COPP).pdf]

# Competence development of Practical Procedures (COPP)

| Preparation and planning | | Excellent completed | Partially completed | Missing | Comments |
|---|---|---|---|---|---|
| **Preparing the patient** | Hand hygiene | | | | |
| | Introduces itself | | | | |
| | Informing about the procedure (necessary, clearly, understandable) | | | | |
| | Consider use of an assistant | | | | |
| **Preparation of equipment, workplace and patient** | Hand hygiene | | | | |
| | Find right equipment and bring it with you in a tray. | | | | |
| | Check durability | | | | |
| | Sprite worktable and organize equipment | | | | |
| | Correct ergonomic position | | | | |
| | Correct position of the patient | | | | |
| | Shield patient/ Not expose the patient. | | | | |
| **Performing** | | | | | |
| **Principles of hygiene** | Proper hand hygiene : in and out of the room/gloves | | | | |
| | Use gloves (sterile / nonsterile), plastic apron, face mask if necessary) | | | | |
| | Following triangle principle of hygiene | | | | |
| | Selects sterile / clean / aseptic method of procedure | | | | |
| | Correct handling of garbage and dirty laundry (laundry bag, garbage bag, sharps) | | | | |
| **Performing procedure according to updated guidelines** | Follows a logically and professionally acceptable order (correct/ precise / accurate) | | | | |
| | Ensure patient safety (Absolute requirements: sterile principles, accurate measurements) | | | | |
| **Supplementary work** | | | | | |
| **Complementation of work and documentation** | Correct position of the patient, paving bed and give the patient bell. | | | | |
| | Clean table and room | | | | |
| | Correct and accurate documentation | | | | |

Assistant professor Elisabeth Solheim and Associate professor Hilde Plathe, USN

| Overall assessment | | Excellent completed | Partially completed | Missing | Comments |
|---|---|---|---|---|---|
| **Fluence** | Without hesitancy | | | | |
| | Without unnecessary breaks | | | | |
| | With ease | | | | |
| **Caring compartment** | Acknowledge | | | | |
| | Show respect | | | | |
| | Ensure patient participation | | | | |
| | Be empatic | | | | |
| | Use appropiate touch | | | | |
| | Be engaged | | | | |
| | Use appropiate communication | | | | |
| | Work aesthetically | | | | |
| | Eyecontact | | | | |

| Knowledge of clinical skill | | Excellent | Less excellent | Missing | Comments |
|---|---|---|---|---|---|
| **Indications / purpose for the procedure** | -Justifying why<br>- Descriptions how<br>- Assessment | | | | |
| **Complications that may arise** | - That threaten patient safety<br>- When using equipment | | | | |
| **Observations** | - Patient and equipment | | | | |
| **Documentation** | - Patient reactions<br>- Observation<br>- Action<br>- Deviation | | | | |
| **Ethical challenges** | - Legal aspect<br>- Ethical aspect | | | | |

Assistant professor Elisabeth Solheim and Associate professor Hilde Plathe, USN
