# Supplementary material for: Nursing Students' and Preceptors' Experiences with Using an Assessment Tool for Feedback and Reflection in Supervision of Clinical Skills: A Qualitative Pilot Study
Source: Nurs Res Pract. 2021 May 18;2021:5551662. doi: 10.1155/2021/5551662 (PMC8154278; doi:10.1155/2021/5551662)
Supplement: Supplementary Materials. — Appendix 1: the assessment tool Competence Development of Practical Procedures (COPPs). Appendix 2: questionnaire to preceptors and students. [file 5551662.f1.zip › 5551662.f1/Appendix 2, questionnaire.pdf]

Appendix 2

Questions—Preceptor Clinical skill: …………………………

Gender (circle): M F

Age (circle): 20–25 26–30 31–35 36–40 41–45 50–55 over 55

In what year did you graduate as a nurse?

List all additional education you have and year of graduation:

What pedagogical training do you have (school/year)?

How many times have you supervised nursing students?
(circle): 0 1–2 3–5 6–10 11–20 over 20 times

1. What were your experiences using COPP to provide supervision of clinical skills?

2. The criteria in COPP are subdivided into three categories (excellent, partially completed and missing) and comments. Was this effective in generating good feedback?

3. What other criteria did you use when assessing the student?

4. If the COPP criteria were not useful, how can they be improved?

5. How was COPP used for analysis and reflection during supervision and evaluation?

6. How was COPP used to link theory/knowledge to clinical skills during evaluation?

7. How was COPP used during evaluation to identify what the student needed to improve (generating feedback)?

8. How can COPP be improved as a formative assessment of students' clinical skills? Any suggestions?

Questions—Student Clinical skill: ……………………………

Gender (circle): M F

Age (circle): 20–25 26–30 31–35 36–40 41–45 50–55 over 55

List all prior education you have and year of graduation:

1. What were your experiences with COPP when self-evaluating clinical skills?

2. The criteria in COPP are subdivided into three categories (excellent, partially completed and missing) and comments. Was this helpful feedback for you?

3. What other criteria, if any, were you evaluated by?

4. If the COPP criteria were not useful, how can they be improved?

5. How did you and the preceptor use COPP to analyse and reflect during evaluation?

6. How did you and the preceptor use COPP to link theory/knowledge to clinical skills during evaluation?

7. Did you experience supervision via COPP to provide helpful feedback on what you need to improve?

8. How can COPP be improved, as a formative assessment of students' clinical skills? Any suggestions?
